# Managers’ and providers’ perspectives on barriers and facilitators for the implementation of differentiated service delivery models for HIV treatment in Mozambique: a qualitative study

**DOI:** 10.1002/jia2.26076

**Published:** 2023-03-14

**Authors:** Dorlim Moiana Uetela, Sarah Gimbel, Celso Inguane, Onei Uetela, Aneth Dinis, Aleny Couto, Irénio Gaspar, Eduardo S. Gudo, Sérgio Chicumbe, Sandra Gaveta, Orvalho Augusto, Kenneth Sherr

**Affiliations:** ^1^ Instituto Nacional de Saúde Maputo Mozambique; ^2^ Department of Global Health University of Washington Seattle Washington USA; ^3^ Department of Child, Family and Population Health Nursing University of Washington Seattle Washington USA; ^4^ National Directorate of Public Health Ministry of Health Maputo Mozambique; ^5^ National STI‐HIV/AIDS Program Ministry of Health Maputo Mozambique; ^6^ Universidade Eduardo Mondlane Maputo Mozambique; ^7^ Department of Epidemiology University of Washington Seattle Washington USA; ^8^ Department of Industrial & Systems Engineering University of Washington Seattle Washington USA

**Keywords:** barriers and facilitators, CFIR, differentiated service delivery, HIV, managers’ and providers’ perspectives, Mozambique

## Abstract

**Introduction:**

In 2018, Mozambique's Ministry of Health launched a guideline for a nationwide implementation of eight differentiated service delivery models to optimize HIV service delivery and achieve universal coverage of HIV care and treatment. The models were (1) Fast‐track, (2) Three‐month Antiretrovirals Dispensing, (3) Community Antiretroviral Therapy Groups, (4) Adherence Clubs, (5) Family‐approach, and three one‐stop shop models for (6) Tuberculosis, (7) Maternal and Child Health, and (8) Adolescent‐friendly Health Services. This study identified drivers of implementation success and failure across these differentiated service delivery models.

**Methods:**

Twenty in‐depth individual interviews were conducted with managers and providers from the Ministry of Health and implementing partners from all levels of the health system between July and September 2021. National‐level participants were based in the capital city of Maputo, and participants at provincial, district and health facility levels were from Sofala province, a purposively selected setting. The Consolidated Framework for Implementation Research (CFIR) guided data collection and thematic analysis. Deductively selected constructs were assessed while allowing for additional themes to emerge inductively.

**Results:**

The CFIR constructs of Relative Advantage, Complexity, Patient Needs and Resources, and Reflecting and Evaluating were identified as drivers of implementation, whereas Available Resources and Access to Knowledge and Information were identified as barriers. Fast‐track and Three‐month Antiretrovirals Dispensing models were deemed easier to implement and more effective in reducing workload. Adherence Clubs and Community Antiretroviral Therapy Groups were believed to be less preferred by clients in urban settings. COVID‐19 (an inductive theme) improved acceptance and uptake of individual differentiated service delivery models that reduced client visits, but it temporarily interrupted the implementation of group models.

**Conclusions:**

This study described important determinants to be addressed or leveraged for the successful implementation of differentiated service delivery models in Mozambique. The models were considered advantageous overall for the health system and clients when compared with the standard of care. However, successful implementation requires resources and ongoing training for frontline providers. COVID‐19 expedited individual models by loosening the inclusion criteria; this experience can be leveraged to optimize the design and implementation of differentiated service delivery models in Mozambique and other countries.

## 1 INTRODUCTION

HIV treatment has been available in Mozambique since 2004 [1] but, despite substantial investment by the country's Ministry of Health (MISAU) and cooperating development partners, the health system is unable to ensure universal coverage of HIV care and treatment for the population [2]. In 2016, the benchmark for success was heightened through the adoption of the then Joint United Nations Programme on HIV/AIDS (UNAIDS) 90‐90‐90 targets (90% of people living with HIV diagnosed, 90% of those diagnosed on treatment and 90% of those on treatment having viral suppression by 2020), and the World Health Organization test and treat strategy that consists of initiation of antiretroviral therapy (ART) for all, regardless of CD4 cell count [3, 4]. To achieve these ambitious targets, innovative strategies to improve the health system's HIV service delivery capacity were required.

In this context, in 2018, MISAU launched a guideline for the nationwide implementation of eight differentiated service delivery (DSD) models for HIV treatment, namely Fast‐track, Three‐month Antiretrovirals (ARVs) Dispensing (3M), Community ART Groups (CAGs), Adherence Clubs (ACs), Family‐approach (FA), One‐stop shop (OSS) for Tuberculosis Services (OSS‐TB), OSS for Maternal and Child Health Services (OSS‐MCH), and OSS for Adolescent‐friendly Health Services (OSS‐AFHS) [5]. DSD is defined as “a client‐centred approach that simplifies and adapts HIV services across the cascade to reflect the preferences, expectations and needs of people living with and vulnerable to HIV, while reducing unnecessary burden on the health system” [6, 7]. The DSD models for HIV treatment are developed based on four building blocks: when, where, who and what. *When* refers to the frequency of service delivery, *Where* refers to the place of service delivery, *Who* refers to the person providing the service and *What* is regarding the services offered [8, 9].

Similar to Mozambique, other countries in sub‐Saharan Africa have adopted DSD models to optimize the health system capacity to offer client‐centred services and improve retention in care, and ultimately reduce HIV‐related deaths [10, 11]. Countries adopt the models that best suit their needs and adapt them to the specific context. Several studies have described the process of DSD models implementation in sub‐Saharan Africa, including acceptability, feasibility and satisfaction, and measured the health and economic impact of different models [10, 11, 12, 13]. Although the body of literature regarding DSD models in sub‐Saharan Africa is growing, general knowledge of the determinants of successful implementation is limited because relatively few studies have explored the determinants of successful implementation and the diversity of the models and various country contexts. Studies that explored the successful implementation of DSD models in specific countries in sub‐Saharan Africa have identified drivers that included the providers’ belief that DSD models were beneficial for both clients and the health system, and the perceived relative advantage of the models when compared with the standard of care, and barriers that included limited availability and access to resources, limited access to training, stigma and challenges with logistics [14, 15, 16, 17]. However, the determinants of the successful implementation of the adopted models in the specific context of Mozambique are yet to be studied.

The goal of our study was to describe managers’ and providers’ perspectives on the determinants of successful implementation of the DSD models adopted in Mozambique, for all models as a package of HIV service delivery and for each specific model, to contribute to the body of knowledge on this subject in sub‐Saharan Africa.

## 2 METHODS

### 2.1 Study setting and period

The study was conducted from July to September 2021. Participants were from MISAU and implementing partners at all levels of Mozambique's health system—national or central, provincial, district and health facility. National‐level data were collected in Maputo city, the country's capital. Sofala province, a setting with high HIV prevalence and HIV treatment demand, including a nationally recognized HIV transmission hotspot—the Beira corridor [18], was purposively selected for data collection at subnational levels. In Sofala province, two districts (one rural and one urban) and four health facilities (one small and one large in each district) were selected. Health facilities are defined by the National STI‐HIV/AIDS Programme as small when they have less than 1000 clients enrolled in HIV treatment services, and as large otherwise.

### 2.2 The DSD models

The eight models being studied are described in Table 1.

**Table 1:** Differentiated service delivery models for HIV treatment implemented in Mozambique

DSD model	What (service offered)	Who (service provider)	When (frequency)	Where (place)	Composition	Particularity
Fast‐track (FT)—Individual model based at facilities	Clinical observation	Clinician	Twice a year	Observation room	Individual	‐ Can be implemented in isolation or combined with 3M. ‐ ARV dispensed quarterly when combined with 3M and monthly in health facilities without 3M.
	ARV dispensing	Pharmacist/pharmacy technician	Monthly/quarterly	Pharmacy		
	Sample collection for lab tests	Laboratory technician	Twice a year	Laboratory		
Three‐Month ART Dispensing (3M)—Individual model based at facilities	ARV pick‐up	Pharmacist/pharmacy technician	Quarterly	Health facility	Individual	Offered only in combination with FT.
Community ART Group (CAG)—Group model managed by clients	Peer support	Client	Monthly	Community	Group (3–6 members)	Members take turns visiting the health facility for clinical observation; all members must be observed and have lab tests done at least twice a year. ARVs for all group members are dispensed monthly to the group member who visits the health facility. This model requires additional staff for activities coordination and implementation.
	Clinical observation	Clinician	Variable	Observation room		
	ARV dispensing	Pharmacist/pharmacy technician	Monthly	Pharmacy		
	Sample collection for lab tests	Laboratory technician	Twice a year	Laboratory		
Adherence Club (AC)—Group model managed by health‐care workers	ART adherence support	Counsellor/peer educator/community health worker	Quarterly	All activities take place in a selected space in the health facility	Group (15–30 members)	ARV dispensing depends on the ARV stock in the health facility. This model requires additional staff for activities coordination and implementation.
	Clinical observation	Nurse	Twice a year			
	ARV dispensing	Nurse	Monthly/quarterly			
	Sample collection for lab tests	Nurse	Twice a year			
Family‐approach (FA)—Group model managed by health‐care workers	Clinical observation	Clinician	Variable	Observation room	Group (varies according to the number of family members)	All the appointments of the family members are schedule for the same day. The frequency of visits depends on the existence and age of children and the clinical condition of all members of the family, and can be monthly, quarterly or twice a year.
	ARV dispensing	Pharmacist/pharmacy technician	Monthly	Pharmacy		
	Sample collection for lab tests	Laboratory technician	Twice a year	Laboratory		
One‐stop shop for TB Services (OSS‐TB)—Individual model based at facilities	Clinical observation	TB sector nurse	Monthly	All activities take place in the TB sector in the health facility	Individual	All HIV services are offered by a nurse in the TB treatment sector of the health facility.
	ARV dispensing	TB sector nurse	Monthly			
	Sample collection for lab tests	TB sector nurse	Twice a year			
One‐stop shop for Maternal and Child Health Services (OSS‐MCH) —Individual model based at facilities	Clinical observation	MCH nurse	Monthly	All activities take place in the MCH sector in the health facility	Individual	All HIV services are offered by a nurse in the MCH sector of the health facility.
	ARV dispensing	MCH nurse	Monthly			
	Sample collection for lab tests	MCH nurse	Monthly			
One‐stop shop for Adolescent‐friendly Health Services (OSS‐AFHS)—Individual model based at facilities	ART adherence support	Counsellor/peer educator/nurse	Quarterly	All activities take place in the AFHS sector in the health facility	Individual	All HIV services are offered in the AFHS sector of the health facility. Clinical observation depends on the client need. ARV dispensing depends on the ARV stock in the health facility.
	Clinical observation	Nurse	Monthly/twice a year			
	ARV dispensing	Nurse	Monthly/quarterly			
	Sample collection for lab tests	Nurse	Twice a year			

### 2.3 Sample size and data collection

We applied purposive sampling to include at least 9–17 key informants so to satisfy the estimated minimum sample size to achieve code saturation of 90% [19, 20]. The eligibility criteria were involvement on DSD models’ management or implementation at each level of the health system, for both MISAU and implementing partners. Semi‐structured, in‐depth interviews were conducted with selected participants, including HIV programme managers from the national, provincial, district and health facility levels, and providers at the health facility level.

The interviews were conducted in Portuguese, using a semi‐structured interview guide that was developed based on purposively selected constructs from the Consolidated Framework for Implementation Research (CFIR) by Damschroder et al. in 2009 [21, 22]. Questions included the perception of barriers and facilitators in general and by selected CFIR constructs, for the intervention overall and for each model individually. Interviews were audio recorded and transcribed verbatim.

CFIR is a deterministic framework developed from previous frameworks and relevant theories in various disciplines [23], and is organized into five domains and 39 constructs (including subconstructs) [21]. We chose to use the CFIR given its pragmatic structure to study real‐world implementation and its applicability to guide data collection and analysis, as well as to contextualize the findings [24].

Constructs for this study were selected based on a literature review of known barriers and facilitators for DSD model implementation in sub‐Saharan Africa. Fifteen constructs from all five framework domains were included: (1) Relative Advantage, (2) Adaptability, (3) Complexity, (4) Design Quality and Packaging, (5) Cost, (6) Intervention Source, (7) Client Needs and Resources, (8) Implementation Climate, (9) Readiness for Implementation, (10) Knowledge and Beliefs About the Intervention, (11) Other Personal Attributes, (12) Planning, (13) Engaging, (14) Executing, and (15) Reflecting and Evaluating [10, 14, 15, 16, 17].

### 2.4 Data analysis

#### 2.4.1 Analysis approach

We conducted a thematic analysis using an iterative deductive–inductive approach. For the deductive analysis, we used an initial list of codes created based on the 15 pre‐selected CFIR constructs. Emerging themes (both non‐CFIR and CFIR constructs) were added to the initial list and used to code subsequent interviews.

#### 2.4.2 Coding description

Coding was conducted on the original interview transcripts in Portuguese, using ATLAS.ti software, version 9. Two investigators (OU and AD) coded each interview transcript independently using the initial codebook and added new codes to it as they emerged from the data. A third investigator (DMU) reviewed the work of the initial coders and identified new codes and disagreements. To achieve consensus on coding, the three investigators reviewed the disagreements and when consensus was not met, two investigators (SG and CI) acted as tiebreakers.

CFIR construct codes were rated as a function of valence and strength. Valence is the directional (positive or negative) influence of the construct on DSD implementation, and is marked by “–” for negative influence (i.e. a barrier), “+” for positive influence (i.e. a facilitator), “X” for mixed negative and positive influence, and “0” for a neutral code. Strength was determined by factors such as the level of agreement across participants, strength of language and use of concrete examples. The number “1” indicated weak influence and “2” strong influence in the intervention. The symbol “*” denoted the level of agreement among participants, meaning that comments were mixed—both positive and negative—and the attributed rating is an aggregated result [25]. Table 2 summarizes the code rating system.

**Table 2:** Coding rating system

Rating	Description
–2	Strong barrier
–1	Weak barrier
–1*	Weak barrier as an aggregated result of positive and negative effect
0	Null meaning
X	Mixed (positive and negative) effect
+1*	Weak facilitator as an aggregated result of positive and negative effect
+1	Weak facilitator
+2	Strong facilitator

### 2.5 Ethics

This study was approved by the Mozambique National Ethics Committee (634/CNBS/20) and the University of Washington institutional review board (FWA#00006878). Written informed consent for interviewing and recording was obtained from interviewees before all interviews. The names of participants and health facilities are concealed for ethical reasons.

## 3 RESULTS

### 3.1 Study participants

We included a total of 20 participants from which 11 were programme managers at the national, provincial and district levels and nine were frontline providers. Four providers (one in each health facility) were also managers at the health facility level. Table 3 shows the participants’ characteristics.

**Table 3:** Overview of study participants

		Sex		Institution	
Level of the health system	Number of participants	Female	Male	MISAU	Implementing partners
National	5	2	3	2	3
Provincial	2	0	2	1	1
District	4	2	2	4	0
Health facility	9	5	4	8	1

### 3.2 Non‐CFIR barriers and facilitators

The identified non‐CFIR‐related determinants were COVID‐19 and Sustainability. COVID‐19 was the most cited non‐CFIR determinant, mentioned across all levels of the health system and by all participants. However, its influence differed depending on the model. Notably, COVID‐19 served as a facilitator for loosening restrictions on client eligibility for DSD models that reduced client visits (FT, 3M), but temporarily interrupted the implementation of group models (AC, CAG). Sustainability was cited as a barrier for AC and CAG models by national‐level managers and frontline providers. Because the implementation of these models requires additional human resources, typically hired by projects from implementing partners, there is no continuity when the projects end. Illustrative quotes related to these determinants are presented in Table 4.

**Table 4:** Illustrative quotes of non‐CFIR barriers and facilitators

Determinant	Illustrative quotes
COVID‐19	*“But if we are to analyse model‐by‐model, we will see that it [COVID‐19] was harmful for CAGs and Adherence clubs, and much more beneficial for the three‐monthly dispensing. All those patients who were on CAGs and Adherence clubs were moved to three‐monthly dispensing.”* (Provincial level)
Sustainability	*“…about the adherence groups, sometimes a project comes and makes them work. But when that project is over, practically those groups will fall apart*.” (Health facility level)

### 3.3 CFIR barriers and facilitators

Across all levels of analysis and across all DSD models, Relative Advantage, Complexity, Patient Needs and Resources, and Reflecting and Evaluating, were the most important CFIR constructs identified. These constructs are described in the sections that follow, along with other constructs, by health system level and DSD model.

### 3.4 Barriers and facilitators by health system level

#### 3.4.1 National level

National‐level managers reported that the currently implemented DSD models were advantageous compared with the standard of care because they were tailored to clients’ needs, were easy to implement, and led to better service and client outcomes. The managers also perceived that the planning process before implementation and continuous monitoring were important facilitators for successful implementation.

Participants at the national level also reported that all models were adaptable to the local context, given that the country selected and designed the models considering the country's needs and resources. The changes made in the DSD models to respond to the COVID‐19 pandemic were also seen as the models’ adaptability. Although participants considered adaptation a facilitating factor at the national level, they did not expect lower levels of the health system to make adaptations to the models.

MISAU's and implementing partners’ managers had different perceptions about the role of external policies and incentives and external change agents. MISAU managers identified these constructs as facilitators for the models that were supported and funded by external organizations, but as barriers for the models without this support, as lack of funding and incentives discourages their implementation, even if providers believe that these models are beneficial for clients. They perceived that implementing partners favour the implementation of some models over others, thus limiting the range of models being implemented in the health facilities they supported. Implementing partners’ managers perceived those constructs as facilitators for DSD models in general, given that there is global support for the implementation of DSD models. However, they mentioned that some models are cheaper than others.

#### 3.4.2 Provincial and district levels

Participants at the provincial and district levels identified the same determinants as those at the national level, except for adaptability as a facilitator and external change agents as a barrier. They considered adaptability to be neutral because they were not supposed to adapt the models but were expected to implement them as recommended by the national level. At these levels, they emphasized planning, readiness for implementation and provider training as important facilitators.

#### 3.4.3 Health facility level

At the health facility level, the main barrier identified was lack of training and resources for both small and large, and rural and urban health facilities. With a few exceptions, frontline providers affirmed that they were not trained to implement the DSD models. They explained that there was a lack of resources for HIV treatment services in general, including for all DSD models, and insufficient human resources for models that required dedicated staff (AC, CAG). In the small health facilities, in addition to a lack of human resources and materials, the lack of adequate space was the main challenge to implementing AC, OSS‐AFHS and OSS‐TB. For the 3M model, stock‐outs of ARVs were identified as a weak barrier, given that stock‐outs were less frequent than they had been at the beginning of the implementation of DSD models. However, participants described a recent stock‐out of ARVs packages for monthly dispensing, which forced the pharmacy to dispense ARVs for 3 months even for clients not enrolled in the 3M model, because the 3‐month packages were the only ARVs available.

Providers perceived that DSD models were beneficial because they reduced their workload. They also believed that the models addressed client needs, based on their assumption that the treatment programme used in DSD models was less demanding and hence reduced the time and resources clients invest in treatment, ultimately allowing clients to engage with other activities. Providers believed that a vital need for clients was related to addressing HIV stigma, which affected clients’ adherence to the treatment in general, including the DSD models. Therefore, they considered that another major benefit of the FT, 3M and FA models was the reduction in the number of visits to health facilities because it would reduce the stigma associated with HIV treatment, as others would not think that the clients were in HIV treatment if they visited the health facility only a few times a year. However, they believed that the downside was that clients who had not yet disclosed their HIV status, due to fear of being stigmatized, were afraid to take home the ARVs for 3 months, because it would be harder to hide from household members. Providers in urban settings believed that clients in urban areas were afraid to share their HIV status with others due to fear of stigma, and thus avoided group models, making these models inappropriate for the urban context.

Finally, participants from all health facilities perceived monitoring and evaluation as an important facilitator for the successful implementation of the models, although they thought that using a paper‐based system was inefficient. They also perceived that all models were relatively easy to implement when compared to the standard of care, especially FT and 3M. CAG and AC were considered more complex among the DSD models; however, they did not consider their relative complexity as a barrier. Illustrative quotes of determinants by CFIR constructs are presented in Table 5.

**Table 5:** Illustrative quotes of barriers and facilitators by CFIR constructs

Determinants by CFIR constructs	Illustrative quotes
Relative Advantage	
Facilitator	*“Compared to the model used previously there are many advantages. For example, there is no overload for the staff working at the district service level, this is the first point. Second, there isn't much crowding at the program level.”* (District level)
Adaptability	
Facilitator	*“The models are adaptable to the local context, so much that the strongest proof is the simplification that we have made in the criteria for the three‐monthly dispensing of ARVs] [in the COVID‐19 context].”* (National level)
Patient Needs and Resources	
Facilitator	*“I believe that the range of options has become increasingly variable, and they are often adjusted to the needs of or the context in which the client is, rural context or other.”* (National level)
Structural Characteristics	
Barrier	*“The health system has different levels, and we have a very high turnover of personnel. So, what happens is that many times, we can train a certain group of providers, but then, six months or a year later, the [trained] provider may no longer be in the same health facility.”* (National level P3)
Available Resources	
Barrier	*“We don't have a physical space for AFHS [Adolescent‐friendly Health Services], and we don't have clinical staff for the same. Regarding 1‐stop shop for tuberculosis, I can say the same as for AFHS; we do not have a physical space specifically for tuberculosis services.”* (Health facility level P17)
Access to Knowledge and Information	
Facilitator	*“We carried out training at the level of health centres, so that each clinician, each MCH [maternal and child health] nurse, who follows up clients, can have more or less what is … the use or implementation of the same models at the level of health facilities.”* (District level P9)
Barrier	*“So to say that the team that is here at the health facility was not trained on differentiated service delivery models.”* (Health facility level)
Knowledge and Beliefs About the Intervention	
Facilitator	*“Honestly, I think it's even better, because it made our activities a little more dynamic, right? Especially the fast‐track for example, it helped a lot.”* (Health facility level P13)
Planning	
Facilitator	*“The guidelines were made, the circulars sent to the provinces, the training on DSD were also done in advance before its implementation. So, I think that [planning] was a facilitator.”* (National level P5)
External Change Agents	
Barrier and Facilitator	*“In the same way that the implementing partners’ interest is a facilitator, it is also a barrier. If the partner is interested in GAG, he will turn his back on the other models, saying, ‘Okay, fine, do it, but that's not what I'm interested in.’”* (National level P2)
Reflecting and Evaluating	
Facilitator	*“So, we are constantly monitoring this, we and the districts that are implementing it, to see what difficulties they are encountering and together we can work out some strategies to overcome them.”* (National level P8)
Barrier	*“Monitoring having mainly paper systems where you have to register all the information to be monitored and you lose a lot of information, and this often harms in terms of quantification and consumption, so here all you have to guarantee is that the models are [registered] in prepared systems and you don't often have these prepared systems.”* (National level P1)

#### 3.4.4 Barriers and facilitators by CFIR construct and DSD model

When asked about the DSD models in general, managers at higher levels of the health system tended to mention a wider list of DSD models before being asked specifically about each of the eight models that we intended to study. At the health facility level, providers primarily mentioned the 3M model, unless another specific model was explored. We were informed that more than 80% of clients using DSD models were enrolled in FT and 3M (3M is only implemented in combination with FT). Since the data collection at the health facilities was done in July 2021, we explored if this scenario resulted from the changes made in the context of COVID‐19, given that in response to COVID‐19, the eligibility criteria for 3M and FT were loosened and the AC and CASG models were temporarily interrupted. According to the participants, although COVID‐19 favoured FT and 3M, these models were the most implemented even before the pandemic, not only because they were considered the easiest to implement among all the models, but also because they were believed to significantly reduce the workload and were largely supported by implementing partners. In Table 6, we present a summary of the determinants of DSD model implementation as aggregated ratings of CFIR constructs by model.

**Table 6:** Aggregated ratings of CFIR constructs by DSD model

	Differentiated service delivery models							
CFIR constructs	FT	3M	AC	CAG	FA	OSS‐MCH	OSS‐AFHS	OSS‐TB
Intervention Characteristic								
Relative Advantage	+2	+2	+2	+2	+2	+2	+2	+2
Adaptability	+1*	+1*	+1*	+1*	+1*	+1*	+1*	+1*
Complexity	+2	+2	+1*	+1*	+1*	+2	+2	+2
Cost	+2	+2	–1	–1	+2	+2	+2	+2
Outer Setting								
Patient Needs and Resources	+2	+2	+1*	X	+2	+2	+2	+2
External Police and Incentives	+2	+2	–1*	–1*	0	0	0	0
Inner Setting								
Structural Characteristics	0	0	–1	–1	–1	–1	–1	–1
Networks and Communications	+1	+1	+1	+1	+1	+1	+1	+1
Culture	+1	+1	+1	+1	+1	+1	+1	+1
Implementation Climate								
Tension for Change	+1	+1	+1	+1	+1	+1	+1	+1
Compatibility	0	0	0	–1*	0	0	0	0
Relative Priority	+2	+2	–1	–1	+1	+1	+1	+1
Goals and Feedback	+1	+1	+1	+1	+1	+1	+1	+1
Readiness for Implementation								
Available Resources	+2	+1*	–2	–1*	0	–1	–2	–1
Access to Knowledge and Information	X	X	X	X	X	X	X	X
Characteristics of Individuals								
Knowledge and Beliefs About the Intervention	+1*	+1*	+2	–1*	+2	+2	+2	+2
Other Personal Attributes	X	X	X	X	X	X	X	X
Process								
Planning	+1	+1	+1	+1	+1	+1	+1	+1
External Change Agents	+2	+2	–1*	–1*	0	0	0	0
Executing	+1*	+1*	–2	–2	+1*	+2	+1*	+1*
Reflecting and Evaluating	+1*	+1*	+1*	+1*	+1*	+1*	+1*	+1*

–2, strong barrier; –1, weak barrier; –1*, weak barrier as an aggregated result of positive and negative effect; 0, null meaning; X, mixed (positive and negative) effect; +1*, weak facilitator as an aggregated result of positive and negative effect; +1, weak facilitator; +2, strong facilitator.

**Abbreviations**: 3M, Three‐month ARV Dispensing; AC, Adherence Clubs; CAG, Community Adherence Group, FA, Family‐approach; FT, Fast‐track; OSS‐AFHS, One‐stop shop model for Adolescent‐friendly Health Services; OSS‐MCH, One‐stop shop model for Maternal and Child Health Services; OSS‐TB, One‐stop shop model for Tuberculosis Treatment Services.

## 4 DISCUSSION

Our study is the first of its kind to explore barriers and facilitators for the implementation of DSD models for HIV treatment in Mozambique, as perceived by managers and providers. The use of a standardized implementation science framework (i.e. CFIR) and of an iterative deductive‐inductive approach, as well as the inclusion of managers and providers from all levels of the health system at MISAU and implementing partner organizations, enabled us to gather strong data on the determinants of successful implementation of DSD models in Mozambique.

The two non‐CFIR determinants identified in the study were COVID‐19 and Sustainability. The overall impact of COVID‐19 was perceived differently among participants. Some understood its influence as neutral given that clients enrolled in models that experienced interruptions were transferred to FT and 3M models. However, other providers reported that the general impact of COVID‐19 was positive because it resulted in the eligibility criteria for enrolment in FT and 3M to be loosened, which allowed more clients to be enrolled in those models. Sustainability was identified as a barrier for AC and CAG as they require additional staff for group management, that are commonly hired for this purpose by implementing partners through projects with limited lifetime, given that MISAU's providers are normally overworked due to a lack of human resources [26]. When the projects are over, the groups are discontinued for lack of a group manager. Sustainability was identified as an important non‐CFIR barrier, based on the CFIR proposed by Damschroder et al. in 2009 [21]. However, in 2020, Means et al. proposed the addition of a new domain to CFIR, the Characteristic of Systems, under which Sustainability could be coded as a Resource Continuity construct [24].

The most influential determinants of successful implementation from a CFIR‐guided lens were the constructs of Relative Advantage, Complexity, Patient Needs and Resources, and Reflecting and Evaluating, which were identified as facilitating factors across all models by participants at all levels of the health system, and Available Resources and Access to Knowledge and Information, identified as barriers at the health facility level. Our findings were consistent with those of other studies on barriers and facilitators of DSD implementation in similar settings [14, 15, 16, 17].

In a study conducted from 2016 to 2017 in South Africa, which included healthcare providers, Department of Health and implementing partners, and three groups of HIV clients (new, established and those not established on treatment or not adhering to care), Pascoe et al. found that providers reported feeling insufficiently trained and described access to adequate resources for implementation as critical [15]. In our study, frontline providers at health facilities identified lack of training as an important barrier to successful implementation, a perspective that was not shared by managers at district, province and national levels. These differing perspectives regarding Access to Knowledge and Information (including training) may be a reflection of the high turnover of frontline providers that is a reality of the Mozambique health system [26]. Managers at levels higher than health facilities believed that providers offering the DSD models received training, whereas frontline providers reported that previously trained colleagues had been transferred to other health facilities, were new or had been recently transferred from other sectors or health facilities and had not yet received training.

A 2017 study of clients from a tertiary health facility in Ghana identified stigma as an important barrier for community‐based service delivery [16]. Stigma was also described by providers in our study as the main reason for non‐adherence to the CAG model in urban areas. Providers believed that clients in urban contexts preferred individual‐based models that did not require them to disclose their HIV status to others. However, the large amount of ARVs dispensed in the 3M model was also described as potentially challenging for clients who had not disclosed their HIV status to household members.

Zakumumpa et al. explored the perspectives of clients and HIV service managers on barriers to the implementation of differentiated ART service delivery in Uganda in 2019, employing a multi‐level analytical framework that analysed individual‐level factors, health system factors, community factors and context [14]. Among other system‐specific barriers not explored in our study, the study revealed several notable findings. At the individual level, stigma and fear of detachment from health facilities by clients established on ART enrolled in community‐based models were identified as important barriers. At the health system level, constraints included insufficient training of health workers in DSD models and supply chain challenges related to dispensing multi‐month ARVs. ARV stock‐outs were also identified as an important barrier in a study conducted in Uganda in 2021, by Kintu et al. [17]. At the community level, limitations included stigma and insufficient funding for the full operationalization of community drug pick‐up points. In addition, frequent physical address changes among urban clients were reported as a challenge for the implementation of CAGs. The determinants described in our study findings are supported by findings from Zakumumpa et al. Stigma and a lack of resources were also identified by Pascoe et al. and Adjetey et al. Interestingly, in our study and the study by Zakumumpa et al., CAGs (referred by them as client groups of rotating ARVs refill pick‐ups) were described as unsuitable for urban environments; however, the reasons given by participants were different. In our study, ARV supply challenges for 3‐month dispensing were perceived as a weak barrier to implementation, because the problem was infrequent at the time of the study. However, we found that stock‐outs of ARVs packages for monthly dispensing forced the dispensing of 3‐month ARVs for clients not enrolled in the 3M model.

In our study, frontline providers believed that the DSD models were beneficial for clients, and health facilities offered the models despite challenges related to a lack of resources and training. Providers also reported that the implementation of DSD models was not optional, given that the decision was made at the national level and passed down through the provincial and district levels to the health facility level. This top‐down decision‐making process is typical of the vertical hierarchical system that characterizes Mozambique's health system [27]. Although this approach is often considered negative for the implementation of new interventions [28], providers in our study did not consider it a barrier or facilitator, but simply as the *modus operandi* of the health system in Mozambique.

We acknowledge the important role of data collectors and analysts on the findings in qualitative research. Participants were interviewed by a study team totally independent and with no influence over the institutions from which the participants were selected and they were informed that their perspectives would not be judged but used to contribute to the knowledge on determinants of DSD models in the country. The interpretation of the findings presented is shaped by the views of the main data analysts—Mozambican medical doctors and implementation scientists, familiar with the Mozambican health system and the process of health programmes implementation.

Our study has several limitations. First, although we used the levels of the health system as the unit of analysis, the study was not designed to generate data on implementation determinants at the micro, meso and macro level and should not be interpreted as such. Second, though the used approach allowed us to triangulate the perspectives of participants at different levels of the health system, we did not consider participants’ maximum variation regarding some relevant characteristics, such as cadre and years of work. Third, the aggregated rating of barriers and facilitators used is subject to reductionism, even though we used the rating rules recommended by CFIR developers [25]. Finally, although our study focused on the perspectives of managers and providers, we recognize that exploring client's perspectives, descriminated by their sociodemographic characteristics, would be an added value. However, clients' inclusion in this study was hindered by the COVID‐19 safety measures in place at the time of data collection. Future studies should explore clients’ perspectives on the determinants of successful implementation for a more comprehensive understanding of this subject.

## 5 CONCLUSIONS

Our study identified determinants for the successful implementation of DSD models, based on managers’ and providers’ perspectives at all levels of the health system in Mozambique. Although the findings cannot be generalized for all providers in Mozambique, they provide an insightful description of barriers and facilitators to be addressed and leveraged for the successful implementation of DSD models in Mozambique. In general, the implemented models were considered advantageous for both the health system and clients when compared with the standard of care. However, successful implementation requires broadly available resources and ongoing training for frontline providers. In addition, COVID‐19 served as a facilitator by loosening inclusion criteria and expediting access to some DSD models, which could be leveraged to optimize the design and implementation of DSD models in Mozambique and other countries.

## COMPETING INTERESTS

The authors declare no competing interests.

## AUTHORS’ CONTRIBUTIONS

DMU conceptualized the research question, the study design and the analytic strategy. CI contributed substantially to the analytic strategy. DMU, OU and AD performed the analysis with substantial inputs from SG and CI. DMU developed the first draft with creative inputs from SG, CI, OU, AD and KS. All authors provided substantial inputs, reviewed and approved the final version.

## FUNDING

The research reported in this publication was supported by the second edition of the complementary grant for doctoral activities for technicians of Instituto Nacional de Saúde (INS), offered by the government of Flanders and by the African Health Initiative of the Doris Duke Charitable Foundation.

## DISCLAIMER

The content of this work is solely the responsibility of the authors and does not represent the funder's views.

## Data Availability

The data that support the findings of this study are available on request from the corresponding author. The data are not publicly available due to privacy or ethical restrictions.
